# Synthesis and Characterization of Holmium-Doped Iron Oxide Nanoparticles

**DOI:** 10.3390/ma7021155

**Published:** 2014-02-12

**Authors:** Maarten Bloemen, Stefaan Vandendriessche, Vincent Goovaerts, Ward Brullot, Maarten Vanbel, Sophie Carron, Nick Geukens, Tatjana Parac-Vogt, Thierry Verbiest

**Affiliations:** 1 KU Leuven, Department of Chemistry, B-3001 Heverlee, Belgium; E-Mails: stefaan.vandendriessche@fys.kuleuven.be (S.V.); vincent.goovaerts@chem.kuleuven.be (V.G.); ward.brullot@fys.kuleuven.be (W.B.); maarten.vanbel@fys.kuleuven.be (M.V.); sophie.carron@chem.kuleuven.be (S.C.); tatjana.vogt@chem.kuleuven.be (T.P.-V.); thierry.verbiest@fys.kuleuven.be (T.V.); 2 PharmAbs, The KU Leuven Antibody Center, KU Leuven, O&N II, Herestraat 49, Box 824, 3000 Leuven, Belgium; E-Mail: nick.geukens@pharmabs.org

**Keywords:** iron oxide, holmium, Faraday rotation, fluorescence, hybrid thin films

## Abstract

Rare earth atoms exhibit several interesting properties, for example, large magnetic moments and luminescence. Introducing these atoms into a different matrix can lead to a material that shows multiple interesting effects. Holmium atoms were incorporated into an iron oxide nanoparticle and the concentration of the dopant atom was changed in order to determine its influence on the host crystal. Its magnetic and magneto-optical properties were investigated by vibrating sample magnetometry and Faraday rotation measurements. The luminescent characteristics of the material, in solution and incorporated in a polymer thin film, were probed by fluorescence experiments.

## 1. Introduction

For many years, intense scientific research has been performed on iron oxide nanoparticles (NP) such as magnetite and maghemite. These particles are non-toxic and cost-effective and are therefore used in many applications, such as drug delivery, biosensing, magnetic separation, magnetic resonance imaging and as contrast reagents [1–5].

Different methods are available for the production of these NP, including hydrothermal and polyol methods, co-precipitation, laser ablation and thermal decomposition of organic iron precursors [1,6]. Sun *et al.* [7] made high-quality magnetite nanocrystals by thermal decomposition of Fe(III) acetylacetonate in phenyl ether. A similar method published by Park *et al.* [8] uses iron-oleate as precursor and oleic acid as capping agent.

To improve the magnetic properties of the NP they can be doped with d-block elements like cobalt, manganese or zinc. Cobalt and manganese both have large magnetic moments, which can increase the saturation magnetization [7,9]. Zinc can occupy the tetrahedral lattice sites, thereby reducing the ferrimagnetism of the magnetite NP [10]. The influence of f-block elements on the crystal’s properties however has not been investigated thoroughly. Rare earth ions are known to have exceptionally large magnetic moments as well as luminescent properties. The latter are shown in photostable upconversion nanoparticles, where energy transfer between two elements (e.g., ytterbium and erbium) results in multicolor luminescence [11]. Another important application of these elements is their usage as a magnetic resonance imaging (MRI) contrast reagent. Gd(III) in particular has seven unpaired electrons which provide excellent contrast in this imaging technique [12,13]. Ho(III) has one of the highest magnetic moments of all elements (10.6 μ_b_) and also exhibits characteristic luminescence.

Another important property of iron oxide NPs is their ability to rotate the polarization of light under the influence of a magnetic field. This so-called Faraday rotation is defined as the rotation of the polarization of light when it passes through a magnetic field in the direction of propagation of light. It is caused by a magnetically-induced difference in the real part of the refractive index for left and right circularly polarized light. It is described by:

θ=VBL (1)

where theta is the measured angle of Faraday rotation, V is the Verdet constant, a wavelength dependent material parameter; *B* is the magnetic field component in the direction of propagation and *L* is the length of propagation in the magnetic field [14,15]. This property is exploited in applications like Faraday isolators and magnetic field sensors [16,17].

In this paper we report the synthesis of luminescent magnetic nanoparticles, by doping iron oxide with different amounts of Ho(III). The magnetic and magneto-optical properties were investigated by vibrating sample magnetometry (VSM) and Faraday rotation measurements. The luminescent properties on the other hand were studied by fluorescence measurements, by both single- and two-photon excitation.

## 2. Results and Discussion

A thorough characterization of the holmium-doped nanoparticles was conducted to investigate the influence of the dopant on the properties of the iron oxide crystal. Four different batches of nanoparticles were prepared having a molar percentage of Ho(III) (compared to iron) of 1.25%, 2.5%, 5% and 10%. The nanoparticles were prepared by thermal decomposition of iron- and holmium oleate precursors, based on the procedure published by Park *et al* [8]. Transmission electron microscopy revealed that the size of the nanocrystals was between 8 and 15 nanometers, with a narrow size distribution, which is a typical result for the synthetic protocol that was followed (TEM images of all batches and exact size distributions can be found in the supporting information, see Table S1 and Figure S1). A more pronounced effect of the holmium doping became visible in X-ray diffraction experiments. A native iron oxide (magnetite, Fe^2+^Fe^3+^_2_O_4_) crystal has an inverse cubic spinel structure. This special type of face-centered cubic crystal lattice has only Fe(III) atoms in the octahedral sites and one Fe(II) and Fe(III) atom in the two tetrahedral sites. A drastic distortion of the crystal can be expected if the atomic radius of the dopant is taken into account. Compared to Fe(III), with a ionic radius of 69 pm, holmium’s radius is 33% larger (104 pm), which introduces local strain in the crystal [18]. This is clearly visible in the peak of the (311) plane, which becomes significantly lower for higher dopant concentrations (data in supporting information, see Figure S2). Higher concentrations of holmium resulted in serious distortion of the crystal structure, and hence a lower count rate. Other planes also show this effect, but less pronounced. We found no evidence that islands of Ho_2_O_3_ are formed within the nanoparticles, even at large holmium doping levels. Figure S3 in the supporting information shows the comparison of the XRD spectra, but no overlap is visible.

The presence of holmium in the nanoparticles was proven further by total reflection X-ray fluorescence (TXRF), which also allowed us to determine the exact concentration of the dopant compared to iron. The determined molar percentage of holmium is only 60% to 80% of the one used in the synthesis, indicating that not all holmium oleate is consumed during the synthesis (see Table 1). Murtaza *et al.* [18] reported that the overall solubility of the dopant in the host’s crystal lattice is low if the ionic radius is not comparable to the host. However, this difference can also be attributed to the possible production of non-magnetic nanoparticles like holmium oxides. These are not retained in the magnetic purification process and hence only serve as a loss of holmium oleate. For clarity, the different batches will be named further according to their nominal holmium content, not the measured concentration.

**Figure 1.**
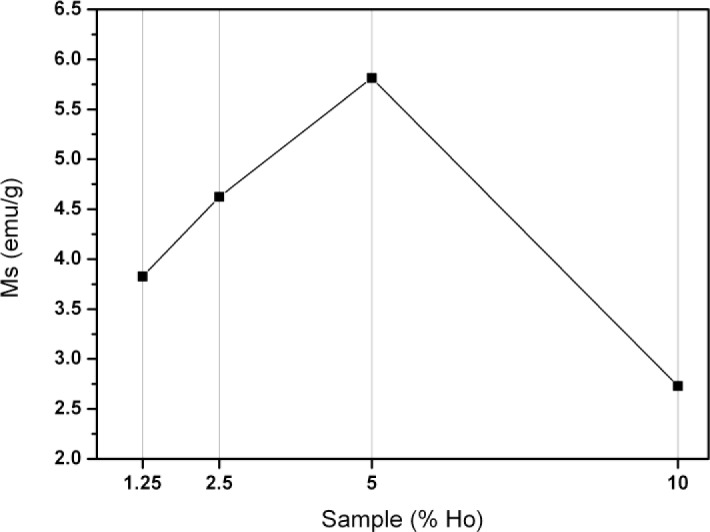
The saturation magnetization of the ferrites is plotted *versus* the molar percentage of holmium(III^+^) in the sample. An optimum is visible for the 5% sample, although the overall magnetization is lower than bulk magnetite. The line, connecting the data points, is solely a guide to the eye.

The magnetic properties of the ferrites were investigated by vibrating sample magnetometry measurements (Figure 1). Although Ho(III) has a high magnetic moment, the resulting nanoparticles have a relatively low saturation magnetization (2.6–6 emu/g) compared to native magnetite (50–60 emu/g). This indicates that the magnetic moments do not accumulate but rather cancel each other out, forming a partially antiferromagnetic material. Incorporation of the dopant into the host crystal structure is probably random. As a result, another likely cause is the loss of the host’s crystal lattice [18]. We hypothesize that the apparent optimum (see Figure 1) can be explained by combining both effects: an initial rise caused by the increasing concentration of holmium and a subsequent loss by distortion of the crystal lattice.

Further results and discussion (Faraday rotation and fluorescence) will focus on the 5% sample, because it has the highest saturation magnetization and hence improved magnetic properties. All data of other samples can be found in the supporting information but are not part of the discussion (see Figures S4–S6).

Introducing these nanoparticles into polymer thin films is a straightforward process that is often used to study their behavior in different environments. Fragouli *et al.* [19] were even able to align the particles under the influence of an external magnetic field. Here, spincoating was chosen to produce the thin films. This technique allows making films of uniform thickness, which is crucial for further experiments. All samples were produced by mixing a solution of poly(methyl methacrylate) (PMMA) in chloroform, with the oleic acid coated NP. After several hours of ultrasonication, these mixtures showed no presence of visible aggregates or precipitation.

**Figure 2.**
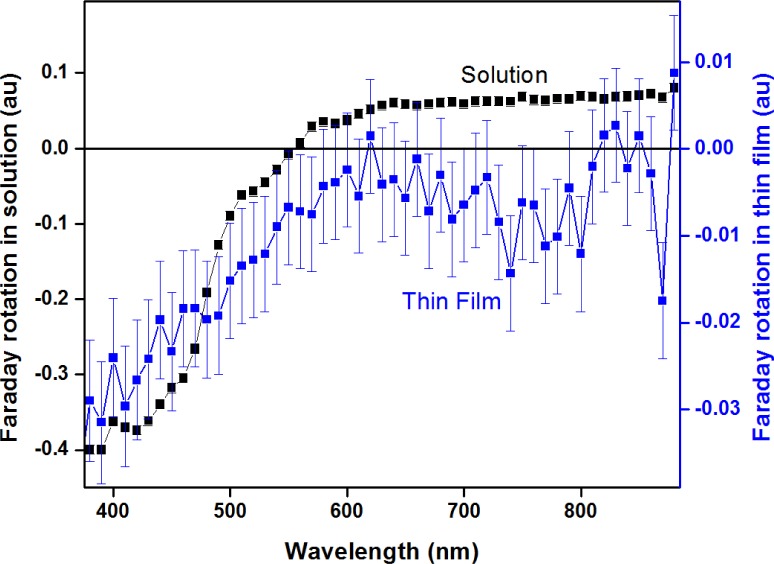
Faraday rotation of the 5% holmium-containing nanoparticles was measured in solution and embedded in a thin polymer film. Although a similar trend is visible in both spectra, the error margins of the thin film are larger, which is caused by uncertainty in the superparamagnetic fitting procedure due to the smaller signal.

When the nanoparticles are subjected to an external magnetic field, along the propagation direction of the light, they exhibit a difference in the real part of the refractive index for left and right circularly polarized light, the so-called Faraday rotation of light. Figure 2 shows the spectrum of the NP in solution and embedded in a PMMA thin film, containing 10 mass percent NP. The spectrum of a solution has a typical shape that corresponds well to the results published by Brullot *et al* [6]. The response of the thin film sample, on the other hand, is smaller, which can be attributed to the smaller thickness of the sample. Since Faraday rotation is related to the length of propagation (Equation (1)) this is an expected result. The oleic acid-coated NP disperse excellent in the apolar polymer, making the thin films uniformly colored and non-scattering, which renders them ideal candidates for Faraday rotation applications.

**Figure 3.**
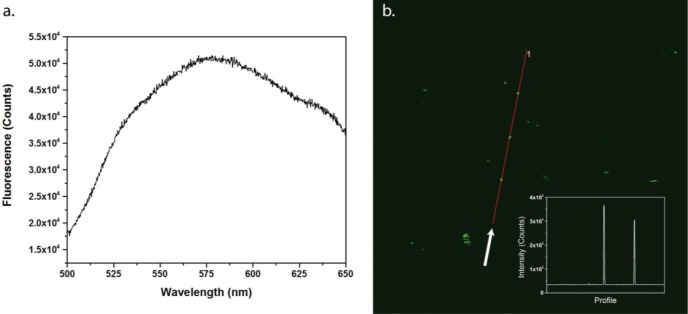
(**a**) Single-photon fluorescence spectrum of the nanoparticles in solution (5 mg/mL), excited at 480nm. The peak can be attributed to the ^5^S_2_,^5^F_4_ ➝ ^5^I_8_ transitions of holmium(III^+^) atoms; (**b**) Two-photon fluorescence image of the nanoparticles embedded in a PMMA thin film. When excited with 850 nm light, the particles emit in the 520–560 nm region, as was also proven in (**a**). An intensity profile (of the red line) is shown as inset in (**b**), to show the presence of aggregates with elevated signal compared to the rest of the film.

As mentioned before, incorporation of the dopant atom into the iron oxide crystal structure also introduces luminescent properties. Luminescence of the nanoparticles was measured by single-photon fluorescence in solution and in the PMMA thin films by two-photon fluorescence. Although iron is well known for its fluorescence quenching properties, the fluorescence was still easily detectable. When the nanoparticles in solution were excited with 480 nm light, a clear signal was detected between 500 and 650 nm, as shown in Figure 3. This luminescence can be attributed to the ^5^S_2_,^5^F_4_ ➝ ^5^I_8_ transitions [20]. The peak broadening that was observed is related to the multiple different environments of the Ho atoms, as well as the iron quenching mechanisms. The thin film sample, on the other hand, was imaged under a microscope by two-photon excitation at 850 nm. Figure 3b shows the signal in the 520–560 nm range of the spectrum. An intensity profile of the red line is shown as an inset. Note that some larger clusters of aggregated nanoparticles were visible under the microscope, showing up as bright dots.

These measurements clearly show that the nanoparticles incorporated in a thin film can be imaged. Overall, the introduction of a dopant into the crystal structure has several interesting effects. Even though the saturation magnetization is lower than for undoped samples, the particles gain fluorescent properties, which could be of great importance for biomedical applications.

## 3. Experimental Section

### 3.1. Synthesis

Ho(III) doped iron oxide nanoparticles were prepared based on the method published by Park *et al.* [8], with small modifications. It consists of two separate reactions, first preparing a holmium- or iron-oleate precursor, which is later transformed into iron oxide nanocrystals.

For the synthesis of the precursor, 36.5 g (120 mmol) sodium oleate and 10.8 g (40 mmol) Fe(III) chloride hexahydrate were dissolved in a mixture of 80 mL ethanol, 60 mL MilliQ water, and 140 mL heptane. This mixture was refluxed at 70 °C for 4 h. Afterward, the upper heptane layer, which contains the iron-oleate, was separated using a separatory funnel and washed three times with 40 mL MilliQ water. As a final step, the heptane was evaporated using a rotavapor, resulting in a dark brown waxy solid. Holmium-oleate was prepared similarly.

Four different ratios of Ho/Fe were prepared: 1.25, 2.5, 5 and 10 molar percentage Ho(III). Typically, the nanoparticle synthesis starts with mixing 0.67 g (0.75 mmol) of the Fe-oleate and 0.071 g (0.075 mmol) of the Ho-oleate with 125.6 microliter of oleic acid and 5 mL of 1-octadecene in a 50 mL three-neck flask. This mixture was first heated to 100 °C for 5 min to evaporate all remaining heptane. After fitting a reflux cooler, the mixture was heated further to 320 °C and kept at that temperature for 30 min. Afterwards the reaction mixture is cooled down to room temperature by removing the heat source. Fifty milliliters of ethanol is added to precipitate the nanoparticles. Separation was done by magnetic separation, after which the particles were washed three times with ethanol. After drying, the nanocrystals were dispersed in toluene in high concentration (100 mg/ml) for long-term storage.

### 3.2. Thin Film Production

Hybrid nanoparticle-polymer films were prepared by mixing 200 mg of polymethylmethacrylate (PMMA, 36,000 g/mol) with 20 mg of nanoparticles in 1ml of chloroform. This mixture was sonicated for 2 h to ensure a homogeneous solution was formed. Thin films were spincoated onto glass slides at 2000 rpm with 2 s of ramp time.

### 3.3. Techniques

Transmission electron microscopy measurements were performed on an 80 kV Zeiss EM-900 (Carl Zeiss AG, Oberkochen, Germany) using 300 mesh Formvar coated copper grids. Distribution data were calculated using ImageJ. Oleic acid coated nanoparticles were dispersed in toluene and deposited onto the grid.

Vibrating Sample Magnetometry experiments were conducted on a VSM Maglab setup from Oxford Instruments (Oxfordshirem, UK).

X-Ray powder diffraction spectra were obtained in reflection (Bragg-Brentano geometry) by a Rigaku Rotaflex diffractometer (Rigaku Corp., Tokyo, Japan), fitted with a Rigaku RU-200B rotating Cu-anode (λ = 1. 54 Å) at a power of 4 kW. The diffracted X-rays were collected after Ni-filtering on a scintillation counter. Samples were deposited on a glass microscope slide from a toluene solution.

In order to measure the Faraday rotation, a laser driven light source (LDLS EQ-99, Energetiq Technology Inc, Woburn, MA, USA) was focused into a collimated beam. After passing through a monochromator, the beam was polarized by a polarizer. A photo-elastic modulator (I/FS50, Hinds Instruments, Hillsboro, OR, USA) at 45 degrees, operating at 50 kHz, then periodically altered the polarization of the beam of light. The beam then passed through a dc magnet, and after passing through an analyzer at 45 degrees with respect to the first polarizer it was detected by a photomultiplier tube. The signal at 100 kHz, corresponding to Faraday rotation, was detected by a lock-in amplifier (SR830, Stanford Research Systems, Sunnyvale, CA, USA). Measurements were performed at discrete magnetic fields.

Metal concentrations were determined with a benchtop total reflection X-ray fluorescence (TXRF) spectrometer (Picofox S2, Bruker, Rheinstetten, Germany). To all the samples a gallium internal standard was added and 20 μL of the solution (100 mg/mL) was brought on the quartz glass sample carriers and dried for 20 min in a hot air oven at 60 °C. The metal concentrations were measured for 500 s and the analysis of the spectra was carried out with the Picofox S2 software.

Steady state luminescence spectra were recorded on an Edinburgh Instruments FS900 steady state spectrofluorimeter (Edinburgh Instruments, Livingston, UK). Quartz cuvettes with 2 mm optical path length were used. Spectra were recorded with a 5 mg/mL solution monitoring the emission between 500 and 650 nm. Excitation of the sample took place at a wavelength of 480 nm.

The two-photon experiments were conducted on a commercial Olympus BX61WI-FV1200-M system (Olympus, Münster, Germany). Detection, in the 520–560 nm range, was carried out non-descanned in backward reflection using a photon counter detector of Hamamatsu R3896 (Hamamatsu Photonics Co., Hamamatsu, Japan). The fundamental wavelength was set to 850 nm (120 fs, 82 MHz pulse rate) on the Mai Tai Deep See laser of Spectra Physics with an average output power of 14 mW on the sample. The polarization of the fundamental beam was controlled using a glan-laser polarizer. The images (512 × 512 pixels) are recorded with a 15× Thorlabs LMV objective (NA of 0.3) and a pixel dwell time of 100 ms.

## 4. Conclusions

We doped iron oxide NP with different amounts of Ho(III) ions and investigated the effect on its properties. A drastic distortion of the crystal structure was observed, even at low concentrations. Similar results were obtained for the saturation magnetization, which was significantly lowered by the incorporation of the dopant, even though an apparent optimum was shown for the 5 molar percent holmium sample. Faraday rotation measurements of the particles showed that the magneto-optical properties were successfully retained if the particles were embedded in a thin film. Finally, the particles’ fluorescence was measured in solution and imaged with a microscope inside the thin film, which clearly showed that the presence of the dopant inside the nanoparticles contributes to its properties. These particles could serve as non-photobleaching probes with a magnetic moment and advanced optical behavior. Embedding them into a polymer thin film retains all these properties, which allowed us to construct a highly functional hybrid material.

## Figures and Tables

**Table 1.** The measured holmium concentration (determined by total reflection X-ray fluorescence (TXRF)) in the iron oxide nanoparticles is lower than the nominal concentration during synthesis.

Sample name	Nominal molar percentage (%)	TXRF molar percentage (%)
Fe_3_O_4_–1.25Ho	1.25	0.92
Fe_3_O_4_–2.5Ho	2.5	2.0
Fe_3_O_4_–5Ho	5	3.8
Fe_3_O_4_–10Ho	10	7.6
