# Supplementary material for: Synthesis and Characterization of Holmium-Doped Iron Oxide Nanoparticles
Source: Materials (Basel). 2014 Feb 12;7(2):1155–64. doi: 10.3390/ma7021155 (PMC5453075; doi:10.3390/ma7021155)

Supporting Information

Table S1. Diameter of the nanoparticles measured by transmission electron microscopy.

Sample name	Nominal holmium content (%)	Diameter (nm)
Fe_3O_4	0	7.24 ± 1.0
Fe_3O_4–1.25Ho	1.25	14.7 ± 1.7
Fe_3O_4–2.5Ho	2.5	11.7 ± 1.0
Fe_3O_4–5Ho	5	8.3 ± 0.7
Fe_3O_4–10Ho	10	10.2 ± 0.8

Figure S1. Transmission electron microscopy images of all different nanoparticle samples.

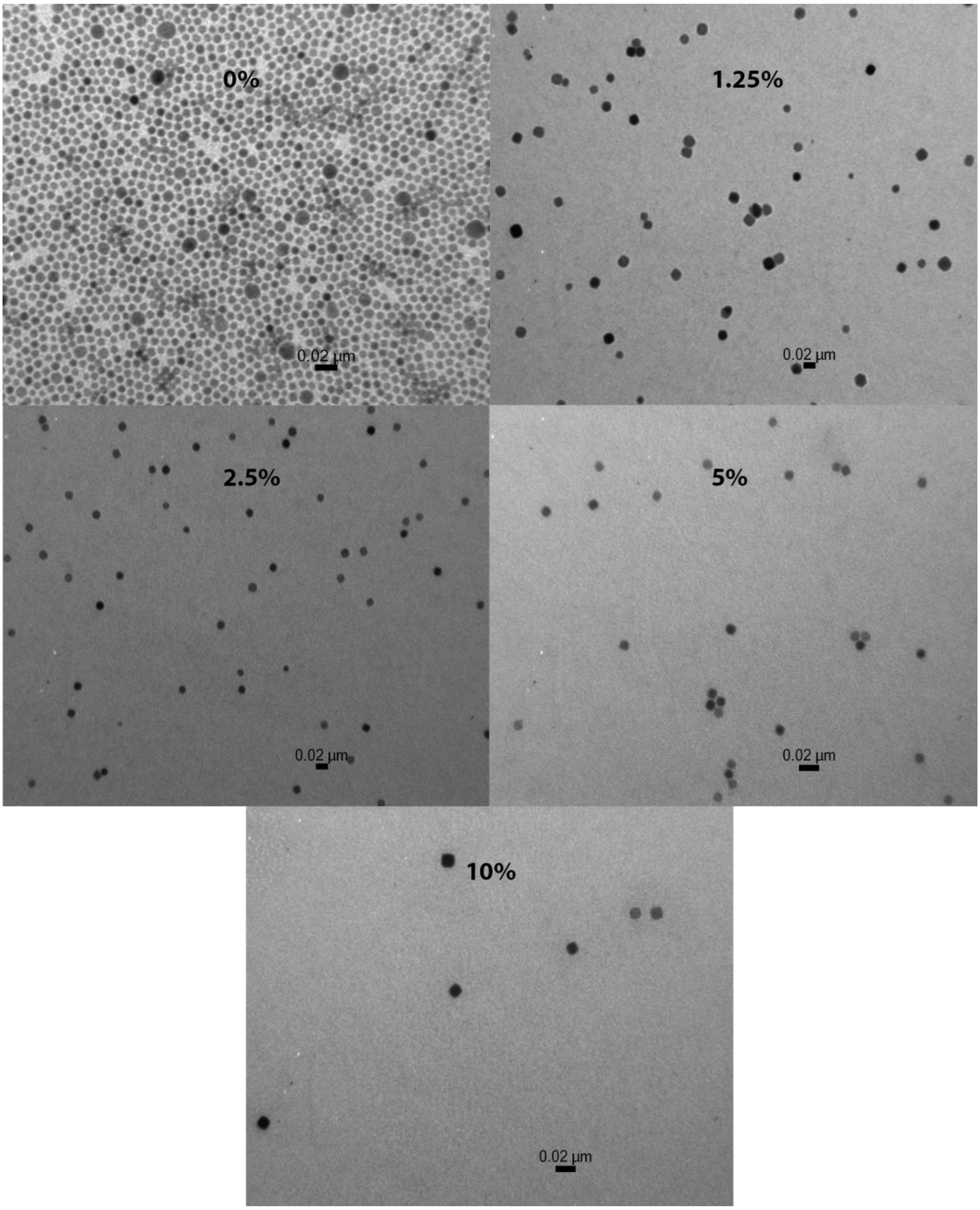

Figure S2. XRD diffraction spectra of the different Ho-doped nanoparticles. The crystal lattice planes are shown next to the corresponding peaks.

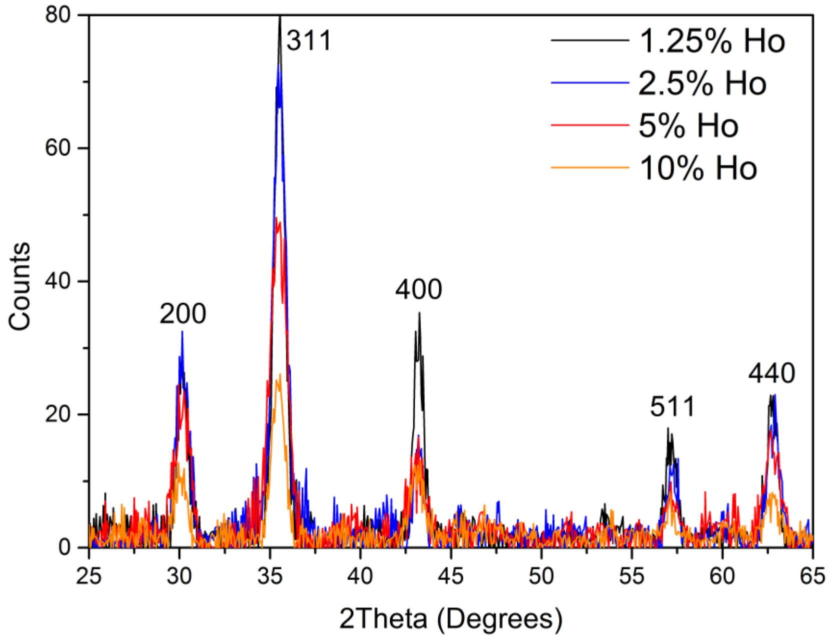

Figure S3. Comparison of the XRD spectra of Ho_2O_3 (COD REV22182 96-210-1513) (red drop-lines) and the 10% Ho-doped sample**.** No overlap is visible, indicating that no islands of Ho_2O_3 are formed within the nanoparticles. It is possible that pure Ho_2O_3 particles are formed during the synthesis, but these are washed away during the magnetic purification process.

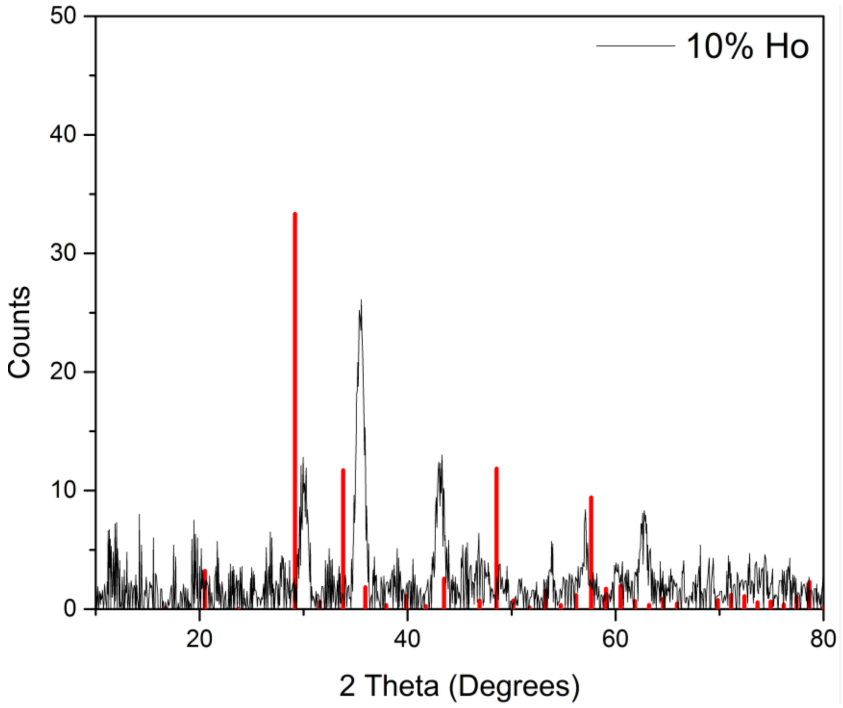

Figure S4. Faraday rotation of the holmium-containing nanoparticles, measured in solution (2.5 mg/mL).

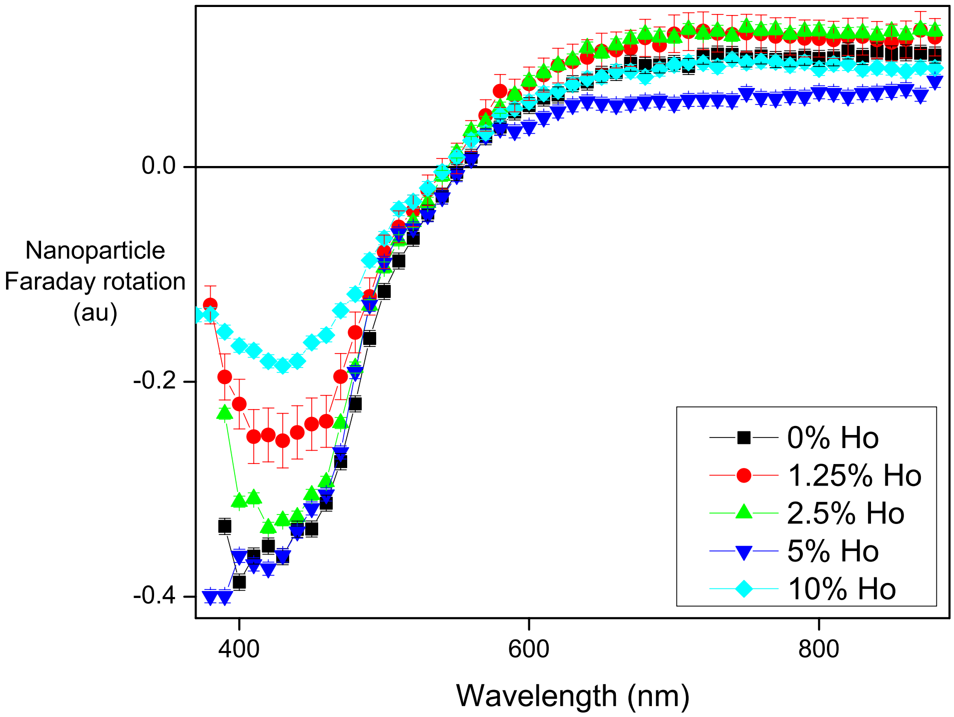

Figure S5. Faraday rotation of the holmium-containing nanoparticles, measured in a thin film. The polymethylmethacrylate film contained 10 mass% particles.

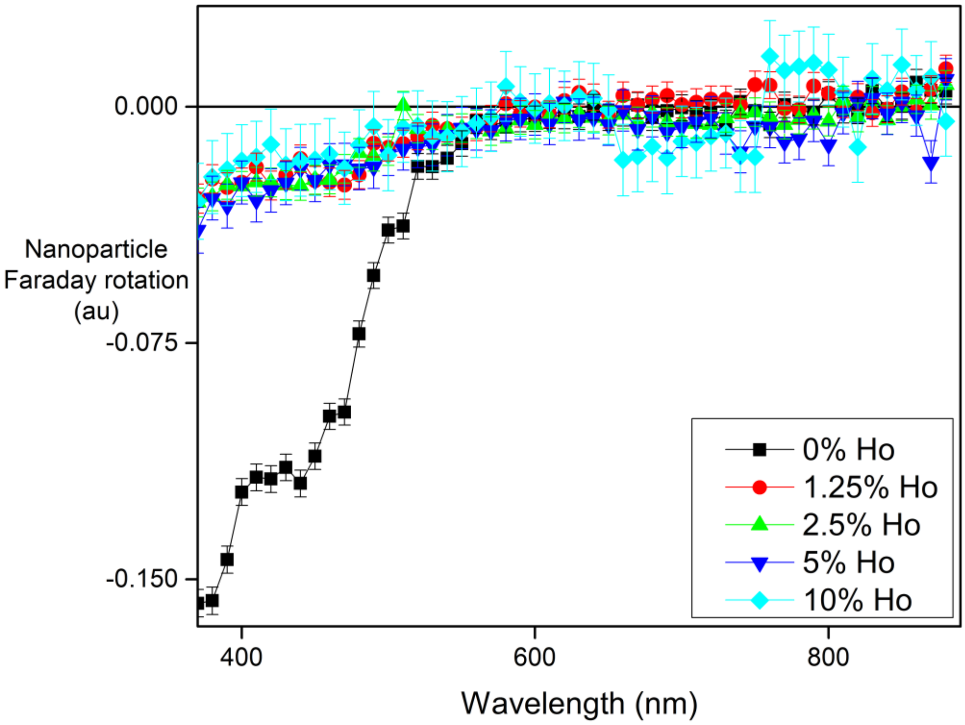

Figure S6. Fluorescence spectra of all nanoparticle samples measured in solution (5 mg/mL, excited at 480 nm). (**a**) 0% Ho; (**b**) 1.25% Ho; (**c**) 2.5% Ho and (**d**) 10% Ho.

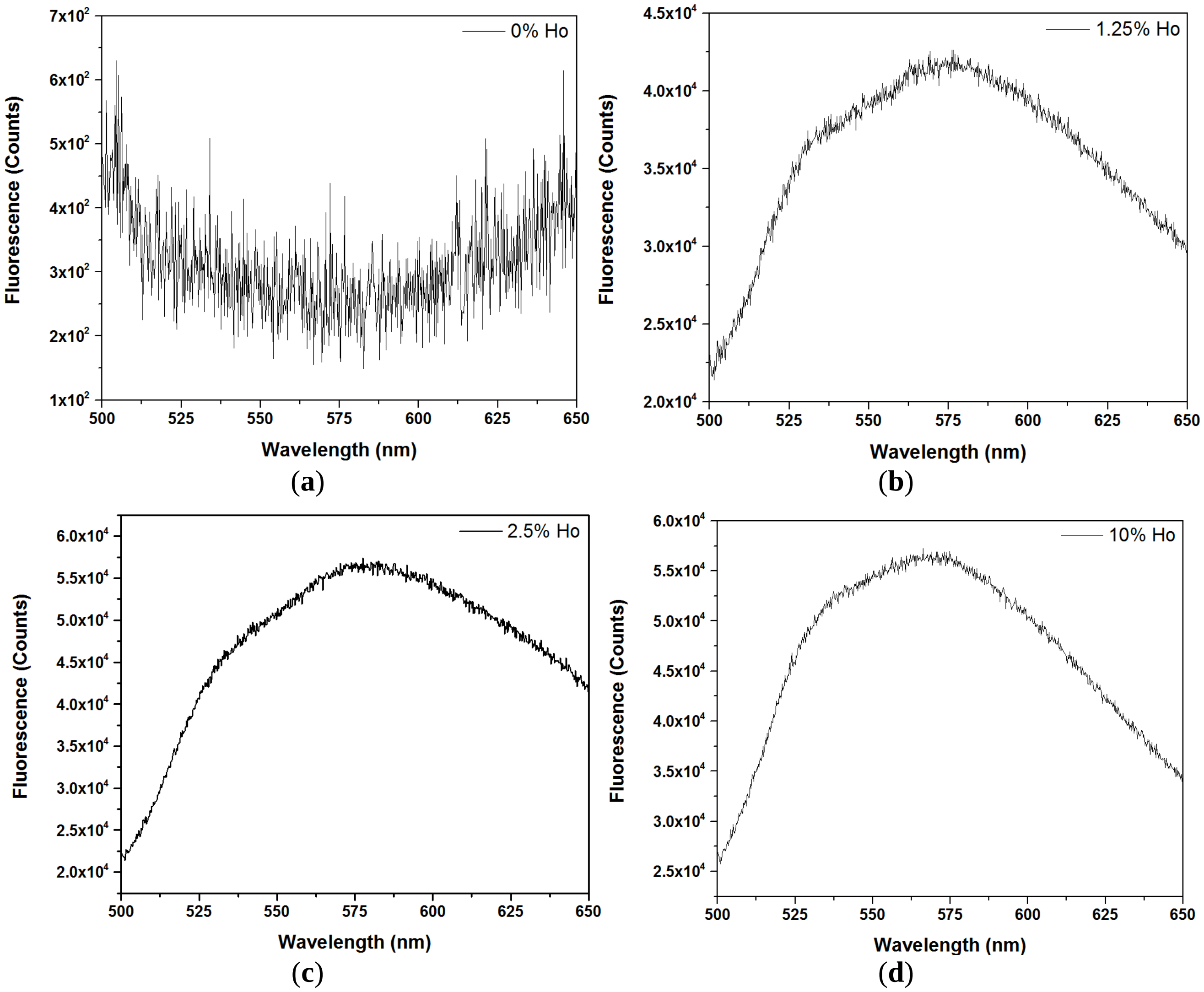

Supplement: Supplementary file 1 [file materials-07-01155-s001.pdf]
